# Estimation of Joint Forces and Moments for the In-Run and Take-Off in Ski Jumping Based on Measurements with Wearable Inertial Sensors

**DOI:** 10.3390/s150511258

**Published:** 2015-05-13

**Authors:** Grega Logar, Marko Munih

**Affiliations:** Faculty of Electrical Engineering, University of Ljubljana, Tržaška cesta 25, 1000 Ljubljana, Slovenia; E-Mail: marko@robo.fe.uni-lj.si

**Keywords:** ski-jumping; force; moments; wearable inertial sensors; Newton, Euler inverse dynamics

## Abstract

This study uses inertial sensors to measure ski jumper kinematics and joint dynamics, which was until now only a part of simulation studies. For subsequent calculation of dynamics in the joints, a link-segment model was developed. The model relies on the recursive Newton–Euler inverse dynamics. This approach allowed the calculation of the ground reaction force at take-off. For the model validation, four ski jumpers from the National Nordic center performed a simulated jump in a laboratory environment on a force platform; in total, 20 jumps were recorded. The results fit well to the reference system, presenting small errors in the mean and standard deviation and small root-mean-square errors. The error is under 12% of the reference value. For field tests, six jumpers participated in the study; in total, 28 jumps were recorded. All of the measured forces and moments were within the range of prior simulated studies. The proposed system was able to indirectly provide the values of forces and moments in the joints of the ski-jumpers' body segments, as well as the ground reaction force during the in-run and take-off phases in comparison to the force platform installed on the table. Kinematics assessment and estimation of dynamics parameters can be applied to jumps from any ski jumping hill.

## 1. Introduction

Ski jumping consists of a complex sequence of movements and can be divided into five main phases according to the biomechanical analysis [1,2]: in-run (IR), take-off (TO), early-flight (EF), stable-flight and landing. Most researchers consider the action during the TO phase as the most important [1–4]. However, the IR phase, which consists of a curved path, is considered equally important, because the athletes actions during the IR determine the initial velocity of TO, the rotating moment and the position of the jumper-ski system during the TO phase [5].

There are two physically-demanding sections of the IR: the curve entrance and the curve exit. In the curve entrance section, the ski jumper maintains an aerodynamic squat position by withstanding the increasing ground reaction force (GRF) and a sudden demand for rotation during the curved part of the hill. The curve exit section requires the opposite effort and is important because it is also near the beginning of the TO phase [5], which is the most critical.

Virmavirta *et al.* [4] directly measured the GRF during TO and showed that the GRF affects the jump length. GRF is equal to the sum of all forces within the body (the result of these forces are the kinetic parameters of each joint segment) minus the gravity force acting on the body. These kinetic parameters can be determined by an analysis of the jump action [6]. The visualization of force patterns over time is an important precursor to understand the cause of a movement. Forces acting within the body cannot be measured directly; however, they can be estimated indirectly from measured kinematic parameters and anthropometric data. The reaction forces, moments and joint moments acting on the athletes' moving joints can be determined through the link-segment model.

Dynamics relates the calculation of forces and moments acting on the body based on available kinematic parameters. Currently, the measurement systems (e.g., force platforms, two- or three-dimensional camera-based system) are limited only to measuring a ski jumping aerodynamic and GRF due to the complexity and availability. Ski jumping aerodynamic forces during the flight phase and GRF during the TO phase have so far been evaluated using a two- or three-dimensional camera-based system [7,8] and a built-in force platform on the table of the ski jumping hill [3,4]. Dynamics, including the calculation of joint forces and the moments of the ski jumper was part of computer simulation studies [5] in past. Due to the force platform and cameras, which have a limited capture area, the dynamics of joints has never been analyzed for the entire ski jump sequence. Force platform- and camera-based systems also require well-trained staff to set up the equipment, and thus, only a few training and research centers worldwide can afford it. Consequently, there is a lack of autonomous systems that can routinely be used to assess the whole ski jump dynamics (*i.e.*, total aerodynamics forces, whole body and each joint dynamics) during the IR, TO and flight phases.

Recently-developed body-worn IMUs are a promising option for determining the kinematic parameters of each joint during a jump. With an IMU-based system, the timing and kinematics during the entire jump sequence have already been calculated [9,10]. Bächlin *et al.* [11] describe the recorded signals, identify characteristic motion patterns and explain bio-mechanically descriptive parameters extracted from accelerometer sensors data. Ohgi *et al.* [12] extracted the aerodynamic force data during jumping flight. Chardonnens *et al.* [13] also designed and assessed a method to determine some aspects of dynamics using IMUs; the position and velocity of the center of mass perpendicular to the table, the force acting on the center of mass perpendicular to the table, somersault angular velocity during TO and the total aerodynamic force during stable flight. Using a miniature, on-body ECG monitor with an integrated acceleration sensor, Kusserow *et al.* [14] assessed the arousal pattern from heart rate data. Based on other studies it might be possible and beneficial to design a simple body-worn IMU-based system that would support the calculation of joint dynamics parameters during IR and TO, as well as GRF during TO, which has never been done for a real ski jump.

This paper presents a method to estimate the forces and moments in the ankle, knee and hip of a ski jumper during the IR and TO phases, as well as a method to estimate the GRF value during the TO phase. The GRF value can be calculated from forces and moments, estimated using the data obtained by a whole body wireless wearable sensory system. In the methodology, the estimation of ski jumper segments' kinematic parameters is described. The dynamic model based on Newton–Euler inverse dynamics and a recursive procedure is presented. The experimental evaluations firstly involving ski jumpers performing jumps in the laboratory and secondly on the ski jumping hill are described in a measurement protocol section. The present work enables innovative, but simple measurements and calculations of joint reaction forces and moments, which provide insight into the body dynamics during IR and TO phases, as well as GRF calculation during the TO phase on any ski jumping hill, regardless of the presence of a built–in force platform.

## 2. Methods

### 2.1. Wearable Measurement System

**Figure 1:**
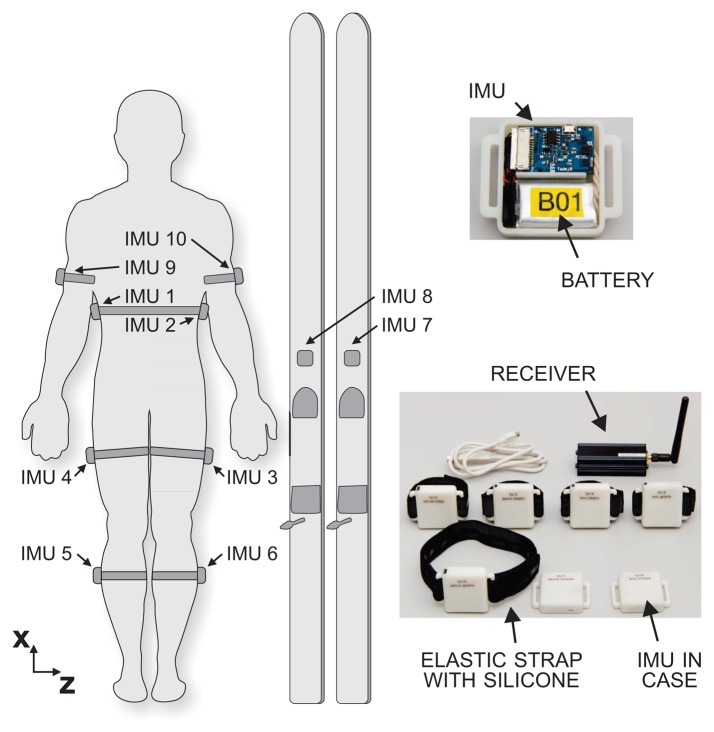
Wearable measurement system composed of 10 inertial measurement units and the setup for positioning the units on the jumper.

The wearable measurement system for ski jumping contains 10 IMUs (Laboratory of Robotics, Faculty of Electrical Engineering, University of Ljubljana; Beravs *et al.* [15]). Each unit contains a 3D accelerometer (±8 g) with a 400-Hz low-pass filter and a 3D gyroscope (±1.000°/s) with a 256-Hz low-pass filter. The signals from each IMU are sampled with a frequency of 400 Hz and stored on a micro-SD card. Two IMUs are attached to the skis in front of the bindings; a further six IMUs are attached to the shanks, thighs and upper arms, and the last two IMUs are affixed to the sacrum of the ski jumper, using dedicated soft elastic straps with a silicone lining to prevent them from slipping. Their positions and the whole system are shown in Figure 1. For synchronization between the units and to indicate the start of the jump, a wireless signal is used. At the end of the workout, the data are wirelessly transferred from the IMUs to a PC for further analysis. A proof of concept was validated in a laboratory environment, where we use for reference measurements an opto-electronic system with tracking markers (Optotrak Certus, Northern Digital Inc., ON, Canada) and a force platform (AMTI OPT464508-1000, Advanced Mechanical Technology, Inc., Watertown, MA, USA). An additional video camera and a built-in force platform at the end of the ski jumping table provide reference measurements of the ski jumping hill tests.

**Figure 2:**
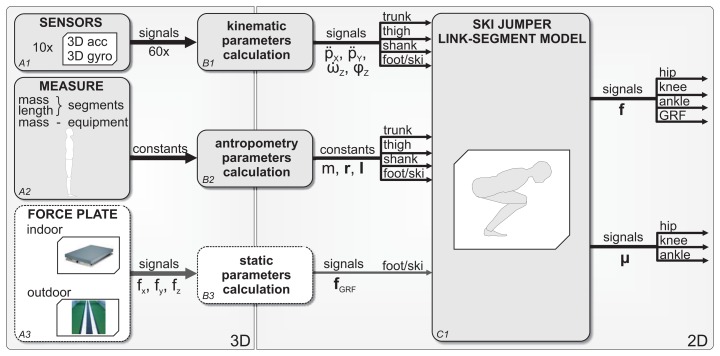
Data flow for measurement data processing. (**A1**) The acceleration and angular velocity of each IMU are obtained; (**A2**) The ski jumpers anthropometric data and equipment are measured; (**A3**) Measuring ground reaction force (GRF) from a force platform; (**B1**) Kinematic and (**B2**) anthropometric parameter calculation; (**C1**) The ski jumper link-segment model calculation.

Measurement data processing is carried out in a systematic order. Figure 2 shows the data flow in steps: (A1) The acceleration and angular velocity are sampled from each IMU at 400 Hz; (A2) The ski jumper's anthropometric data and equipment are measured; (A3) The measured GRF from a force platform is used for validation; (B1) The kinematic and (B2) anthropometric parameters are calculated as explained in the Methods section; (C1) Ski jumping was considered as a planar problem with the assumption of body symmetry; the resulting outputs of the model are joint forces and moments acting in the sagittal plane. The inputs to the model are the linear and angular acceleration (*p̈_x_*,*p̈_y_*,*ω̇_z_*) and orientation (*φ*) of each segment of the jumper. Each segment consists of the mass (*m*), moment of inertia (**I**) and length vector (**r**) from the segment's center of mass (CoM) and the center of the joint. For the validation of the model, the GRF (**f***_GRF_*) is used as the input. Joint forces and moments were calculated for ankles, knees and hips.

### 2.2. Calibration Procedure

When attaching the IMUs onto the jumper, the xyz frames of the IMUs can never be exactly aligned with the proposed “anatomical” XYZ frames of the body (the center of the XYZ frame is at the CoM of the segment; the axes are then: medial-lateral, dorsal-ventral and anterior-posterior). A calibration procedure is carried out to make the system independent of attaching IMUs on the body: first, the jumper is asked to stand still for five seconds. The mean acceleration vector, measured by each IMU while standing in this position, is used to define the inferior-superior axis. Secondly, the jumper is asked to lie down and be still for five seconds. The mean acceleration vector measured by each IMU in this position is used to define the posterior-anterior axis. The body-to-sensor rotation matrix is computed only by means of the accelerometer signals following the procedure mentioned above. The details of the calculation are explained by Palermo *et al.* [16]. This applies a rotation to the sensor signals, in order to express the acceleration and angular velocity relative to the anatomical XYZ frame of the segments. To calculate the acceleration and angular velocity at the segments' CoM, Equations (1) and (2) were used instead of IMUs:

(1) $${\ddot{p}}_{S_{\text{COM}}}={\ddot{p}}_{S_{\text{IMU}}}+\omega_{S_{\text{IMU}}}\times \omega_{S_{\text{IMU}}}\times r_{S_{\text{IMU}\to \text{CoM}}}+{\dot{\omega }}_{S_{\text{IMU}}}\times r_{S_{\text{IMU}\to \text{CoM}}}$$

(2) $$\omega_{S_{\text{COM}}}=\omega_{S_{\text{IMU}}}$$

where *S* stands for the body segment; *ω_S_IMU__* and *ω_S_COM__* for the angular velocity of the segment at the IMU and CoM location; *ω̇_S_IMU__* for the angular acceleration vector of the segment at the IMU location obtained by numerical derivation of *ω_S_IMU__*; **P̈***_S_IMU__* and **P̈***_S_COM__* for the acceleration vector at the IMU and CoM location; and **r***_S_IMU→CoM__* for the vector between the segment IMU and CoM location.

The measured acceleration is a combination of gravitational acceleration, as well as acceleration caused by the jumper's motion, which is explained in the following equation:

(3) $${\ddot{\text{P}}}_{S_{\text{IMU}}}={\ddot{\text{P}}}_{\text{din}}-g_{0}$$

### 2.3. Segment Kinematics

The orientations of the skis, shanks, thighs and HAT (head, arms and trunk) frames relative to the reference ski jumping hill table frame are calculated for the complete duration of each ski jump. First, the initial orientation of each segment is obtained. Secondly, the segment's angular velocity is integrated to obtain the orientation. Thirdly, to obtain the orientation of the segment in the sagittal plane, the sum of the initial orientation and the integrated angular velocity is calculated.

The initial orientation is calculated during the IR, where all of the segments and skis have comparable acceleration. The skis are parallel to the tracks of the jumping hill, and the sagittal inclination of the skis is equal to the inclination of the table. The initial orientation of the segments is calculated step by step according to the orientation of the skis, and the orientation between the segments is calculated using the equation:

(4) $$\cos\varphi_{S\to \text{ski}}=\frac{{\ddot{\text{P}}}_{S}\cdot {\ddot{\text{P}}}_{\text{ski}}}{\left|{\ddot{\text{P}}}_{S}\right|\cdot \left|{\ddot{\text{P}}}_{\text{ski}}\right|}$$

where **p̈***_S_* and **p̈***_ski_* are the vectors of the median acceleration of the segments and the ski, while the jumper is in the straight section during IR, and *φ* represents the angle between the skis and the segment. All of the orientations obtained in the second step, are expressed in terms of the skis, so that the orientation of the slope is added.

Angular velocity integration: Before each jump, the jumper is asked to stand still for 5 s. The mean angular velocity vector, measured by each IMU in this position, is used to define the bias of each axis of the gyroscope sensors. After that, for each IMU's angular velocity signal, the bias is subtracted. The orientation for each segment or ski is then calculated using the numeric trapezoid integration method [17].

### 2.4. Body Measurement and Anthropometric Parameters

For a good estimate of the ski jumper link-segment model, the exact parameters of each segment, such as mass (*m_segment_*), CoM position, length (*l_segment_*) and moment of inertia (**I***_segment_*), are required. In this study, these data were obtained from statistical tables based on the height, weight and gender of the ski jumper [18]. The obtained anthropometric data are recalculated to our simplified model. For *m_hat_*, the sum of all of the masses of the upper body is calculated: *m_thigh_* is the sum of both thighs; *m_shank_* is the sum of both shanks; and *m_ski_* is the sum of both skis and feet. The CoM of the HAT segment is calculated, based on the CoM of each upper body segment. The CoM of thigh segment in the model is calculated based on the CoM of both thighs. The CoM of the shank segment in the model is calculated based on the CoM of both shanks. The CoM of the foot-ski segment in the model is calculated based on the CoM of both skis and feet. The **I***_segment_* for each segment of our model in the sagittal plane is calculated based on the Huygens–Steiner theorem [19]; **I***_HAT_* from each **I** of the upper body, **I***_thigh_* from both **I** of the thigh, **I***_shank_* from both **I** of the shank and **I***_foot-ski_* from each **I** of the skis and feet.

### 2.5. Ski Jumper Link-Segment Model

**Figure 3:**
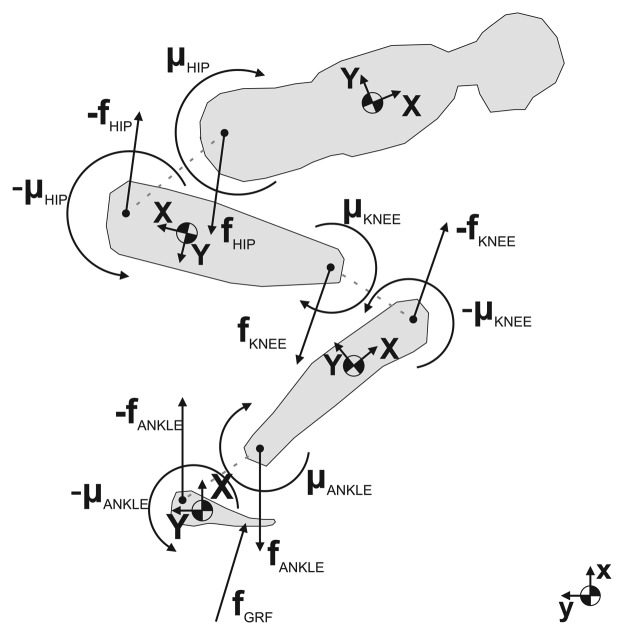
Recursive Newton–Euler inverse dynamics model of a ski jumper. The basic idea of the recursive approach is to first calculate variables for segment *i*, then calculate the unknown joint variables *i* + 1 and, in the next step, proceed with the calculation.

The ski jumper model is defined as a multi-segment system of rigid bodies with constant weights. The segments are interconnected with idealized joints; as a result, each segment is treated as an independent body [20]. The movement of the system is described by the Newton–Euler inverse dynamics analysis. The Equations (5) and (6) and Figure 3 are valid for a dynamics balance, expressed by a local coordinate system of the segment, where **f** and ***μ*** represent the forces and moments acting on each joint.

(5) $$\sum \text{f}=m{\ddot{\text{P}}}_{c}$$

(6) $$\sum \mu =\text{I}\dot{\omega }$$

We do not want to subtract the effect of gravitation from the accelerometer data. Our approach for solving the model is to utilize this effect and to use it to our advantage. Combining the Equations from Equation (1) to Equation (3), we simplify Equations (5) and (6) for the model described in Figure 3. Simplification is used to solve the model without subtraction of gravitational acceleration and is explained with equation:

(7) $$m_{\text{seg}}\left({\ddot{\text{P}}}_{c_{\text{seg}}}-g_{0}\right)=m_{\text{seg}}{\ddot{\text{P}}}_{S_{\text{COM}}}$$

where *m_seg_* is the weight of the segment, ***g***_0_ is gravitational acceleration, **p̈***_cseg_* is the linear acceleration of the center-of-mass of the segment and **p̈**_*S_COM_*_ is the calculated acceleration of the segment's CoM based on Equations (1) and (3). The final model can be described in the equations:

(8) $${\text{f}}_{\text{GRF}}-{\text{f}}_{\text{ankle}}=m_{\text{foot}-\text{ski}}{\ddot{\text{P}}}_{S_{\text{foot}-\text{ski}}}$$

(9) $${\text{f}}_{\text{ankle}}-{\text{f}}_{\text{knee}}=m_{\text{shank}}{\ddot{\text{P}}}_{S_{\text{shank}}}$$

(10) $${\text{f}}_{\text{knee}}-{\text{f}}_{\text{hip}}=m_{\text{thigh}}{\ddot{\text{P}}}_{S_{\text{thigh}}}$$

(11) $${\text{f}}_{\text{hip}}=m_{\text{hat}}{\ddot{\text{P}}}_{S_{\text{hat}}}$$

(12) $$r_{c_{\text{foot}-\text{ski}},\text{GRF}}\times {\text{f}}_{\text{GRF}}-\mu_{\text{ankle}}-r_{c_{\text{foot}-\text{ski}},}\times {\text{f}}_{\text{ankle}}={\text{I}}_{\text{foot}-\text{ski}}{\dot{\omega }}_{\text{foot}-\text{ski}}$$

(13) $$\mu_{\text{ankle}}+r_{c_{\text{shank}},\text{ankle}}\times {\text{f}}_{\text{ankle}}-\mu_{\text{knee}}-r_{c_{\text{shank}},\text{knee}}\times {\text{f}}_{\text{knee}}={\text{I}}_{\text{shank}}{\dot{\omega }}_{\text{shank}}$$

(14) $$\mu_{\text{knee}}+r_{c_{\text{thigh}},\text{knee}}\times {\text{f}}_{\text{knee}}-\mu_{\text{hip}}-I_{c_{\text{thigh}},\text{hip}}\times {\text{f}}_{\text{hip}}={\text{I}}_{\text{thigh}}{\dot{\omega }}_{\text{thigh}}$$

(15) $$\mu_{\text{hip}}+r_{c_{\text{hat}},\text{hip}}\times {\text{f}}_{\text{hip}}={\text{I}}_{\text{hat}}{\dot{\omega }}_{\text{hat}}$$

Descriptions of the parameters in the Equations from Equation (8) to Equation (15) are given in Table 1.

### 2.6. A Recursive Procedure to Calculate the Forces and Moments in the Joints

The Newton-Euler inverse dynamics analysis is based on a recursive procedure to calculate the forces and moments in the joints of a multi-segment structure. The recursive procedure starts at the first segment, which is usually in contact with the surroundings, and continues to the last segment in the chain. The external forces and moments can act on any segment of the chain at any point. The forces need to be known, calculable or measurable. The kinematic chain of the ski jumper is formed by the extremities (left leg, right leg, torso with head and hands). The 2D model is applied for foot-ski, shank, thigh and HAT segments. This approach treats each segment as an independent body [20]. The error in the recursive approach cumulates from the first to the last segment in the chain. To distribute the error evenly, we could solve all of the differential equations simultaneously, but in the recursive approach, that is not possible. To find the smallest cumulated error, three different recursive procedures were implemented to calculate the forces and moments based on the ski jumpers link-segment model: bottom-up, top-down and top-down-up.

#### 2.6.1. Bottom-up Inverse Dynamics

First, a bottom-up inverse dynamics procedure is used for the reference value. Start at the foot-ski segment where the GRF is known (measured with the force platform), and continue calculation to the last segment in the chain. The forces and moments are calculated for the ankle, knee and hip (**f***_ankle_*, ***μ****_ankle_*, **f***_knee_*, ***μ****_knee_*, **f***_hip_* and ***μ****_hip_*) solving the Equations in the order demonstrated below:

1. In Equation (8) **f***_ankle_* is calculated; **f***_GRF_* is measured with force platform; *m_foot−ski_* is constant; **p̈**_*foot-ski_COM_*_ is acquired with IMU. **f***_ankle_* is calculated in segment local coordinate system, for use in the next step should be transformed to global coordinate system.
2. In Equation (9), **f***_knee_* is calculated; **f***_ankle_* is transformed from the global to shank local coordinate system; *m_shank_* is constant; **P̈**_*shank_COM_*_ is acquired with the IMU. **f***_knee_* is calculated in the segment local coordinate system; for use in the next step, it should be transformed to the global coordinate system.
3. In Equation (10), **f***_hip_* is calculated; **f***_knee_* is transformed from the global to thigh local coordinate system; *m_thigh_* is constant; **P̈**_*thigh_COM_*_ is acquired with the IMU. **f***_hip_* is calculated in the segment local coordinate system; for use in the next step, it should be transformed to the global coordinate system.
4. In Equation (12), *μ_ankle_* is calculated; **f***_GRF_* is measured with the force platform; **f***_ankle_* is transformed from the global to foot-ski local coordinate system; **P̈***_foot−ski_* is acquired with the IMU; ***r***_*c_foot-ski_,GRF*_ is calculated from the measured forces and moments at the center of pressure with the force platform; ***r***_*c_foot-ski_,ankle*_ and **I***_foot−ski_* are constants. *μ_ankle_* is calculated in the segment local coordinate system; for use in the next step, it should be transformed to the global coordinate system.
5. In Equation (13), ***μ****_knee_* is calculated; **f***_ankle_*, *μ_ankle_* and **f***_knee_* are transformed from the global to shank local coordinate system; ***ω̇****_shank_* is acquired with the IMU; ***r***_*c_shank_,ankle*_, ***r***_*c_shank_,knee*_ and **I***_shank_* are constants. ***μ****_knee_* is calculated in the segment local coordinate system; for use in the next step, it should be transformed to the global coordinate system.
6. In Equation (14), ***μ****_hip_* is calculated; **f***_knee_*, *μ_knee_* and **f***_hip_* are transformed from the global to thigh local coordinate system; ***ω̇****_thigh_* is acquired with the IMU; ***r***_*c_thigh_,knee*_, ***r***_*c_thigh_,hip*_ and **I***_thigh_* are constants. *μ_hip_* is calculated in the segment local coordinate system; for use in the next step, it should be transformed to the global coordinate system.

#### 2.6.2. Top-down Inverse Dynamics

Secondly, a top-down inverse dynamics procedure is used. It starts at the HAT segment, where the external forces are not known. It is assumed that none of the external forces act on the starting segment of the chain (HAT). The procedure continues to the foot-ski segment (**f***_hip_*, ***μ****_hip_*, **f***_knee_*, *μ_knee_*, **f***_ankle_*, **f***_grf_* and *μ_ankle_*) solving the Equations in the order demonstrated below:

1. In Equation (11), **f***_hip_* is calculated; *m_hat_* is constant; **p̈**_*hat_COM_*_ is acquired with the IMU. **f***_hip_* is calculated in the segment local coordinate system; for use in the next step, it should be transformed to the global coordinate system.
2. In Equation (10), **f***_knee_* is calculated; **f***_hip_* is transformed from the global to thigh local coordinate system; *m_thigh_* is constant; **p̈**_*thigh_COM_*_ is acquired with the IMU. **f***_knee_* is calculated in the segment local coordinate system; for use in the next step, it should be transformed to the global coordinate system.
3. In Equation (9), **f***_ankle_* is calculated; **f***_knee_* is transformed from the global to shank local coordinate system; *m_shank_* is constant; **p̈**_*shank_COM_*_ is acquired with the IMU. **f***_ankle_* is calculated in the segment local coordinate system; for use in the next step, it should be transformed to the global coordinate system.
4. In Equation (15), *μ_hip_* is calculated; **f***_hip_* is transformed from the global to HAT local coordinate system; ***ω̇****_hat_* is acquired with the IMU; ***r****_c_hat_,hip_* and **I***_hat_* are constants. ***μ****_hip_* is calculated in the segment local coordinate system; for use in the next step, it should be transformed to the global coordinate system.
5. In Equation (14), ***μ****_knee_* is calculated; **f***_knee_*, ***μ****_hip_* and **f***_hip_* are transformed from the global to thigh local coordinate system; ***ω̇****_thigh_* is acquired with the IMU; ***r****_c_thigh_,knee_*, ***r****_c_thigh_,hip_* and **I***_thigh_* are constants. ***μ****_knee_* is calculated in the segment local coordinate system; for use in the next step, it should be transformed to the global coordinate system.
6. In Equation (13), ***μ****_ankle_* is calculated; **f***_ankle_*, ***μ****_knee_* and **f***_knee_* are transformed from the global to shank local coordinate system; ***ω̇****_shank_* is acquired with the IMU; ***r****_c_shank_,angle_*, ***r****_c_shank_,knee_* and **I***_shank_* are constants. ***μ****_ankle_* is calculated in the segment local coordinate system; for use in the next step, it should be transformed to the global coordinate system.
7. In Equation (8), **f***_GRF_* is calculated; **f***_ankle_* is transformed from the global to foot-ski local coordinate system; *m_foot−ski_* is constant; **p̈**_*foot-ski_COM_*_ is acquired with the IMU. **f***_GRF_* is calculated in the segment local coordinate system; for use in the next step, it should be transformed to the global coordinate system.

#### 2.6.3. Top-down-up Inverse Dynamics

The third method, a top-down-up inverse dynamics procedure, is a combination of both previously-mentioned methods. The external forces on the foot-ski segment are not measured; it is also assumed that none of the external forces act on the HAT segment. First, the forces are calculated from the segment HAT to the foot-ski segment (**f***_hip_*, **f***_knee_*, **f***_ankle_* and **f***_GRF_*). Second, the moments are calculated in the opposite direction from the foot-ski segment to the HAT segment (***µ****_ankle_*, ***µ***_knee_ and ***µ****_hip_*). The solution of equations is demonstrated below:

1. The calculation of **f***_hip_* is explained in Step 1 of the top-down inverse dynamics procedure.
2. The calculation of **f***_knee_* is explained in Step 2 of the top-down inverse dynamics procedure.
3. The calculation of **f***_ankle_* is explained in Step 3 of the top-down inverse dynamics procedure.
4. The calculation of **f***_GRF_* is explained in Step 7 of the top-down inverse dynamics procedure.
5. The calculation of ***µ****_ankle_* is explained in Step 4 of the bottom-up inverse dynamics procedure with two exceptions: **f***_GRF_* is not measured, but is calculated in the previous step (top-down-up Step 4); ***r***_*c_foot-ski_,GRF*_ is calculated from the end of the toes to the foot's CoM;
6. The calculation of ***µ****_knee_* is explained in Step 5 of the bottom-up inverse dynamics procedure.
7. The calculation of ***µ****_hip_* is explained in Step 6 of the bottom-up inverse dynamics procedure.

### 2.7. Measurement Protocol

For the model validation, four ski jumpers from the Slovenian National Nordic Center Kranj (DPNC Kranj) were enrolled in the study. They had different individual jumping techniques and were 19(4) years old, had a body height of 1.78(6) m and a body mass of 60(7)kg (mean ± standard deviation (SD)). First, they were asked to perform a simulated jump in a laboratory on a force platform (AMTI). In total, 20 jumps were recorded by both the marker-based motion capture reference system (Optotrak) and the IMU-based measurement system, used simultaneously. The IMU-based and reference systems were electronically synchronized by a trigger signal. The entire experiment was filmed with a video camera.

For outdoor validation, the best six ski jumpers (age 18.9(30) years, body height 1.76(3) m and body mass 61.1(23) kg) from a Slovenian ski jumping club (Smučarsko skakalni klub Ilirija – SSK Ilirija) were enrolled in the study. In total, 28 jumps were recorded on a HS-106 (hill size 106 m) jumping hill (Frenštát, CZE) during the summer season. This ski jumping hill has a built-in, 6.41 m-long force platform at the end of the table. The IMU-based and the force platform systems were electronically synchronized by an optical trigger sensor located at the beginning of the force platform. Each jump was filmed with a video camera from the trainer position.

Bottom-up, top-down and top-down-up inverse dynamics procedures were used for the indoor experiment. The top-down-up inverse dynamics procedure, which is based on the findings of the indoor experiment, was used for the outdoor experiment.

The experiments were carried out within the framework of a research program approved by the Slovenian medical ethics committee.

## 3. Results

### 3.1. Indoor Validation Results

Following the previously described recursive procedure, the forces and moments in the joints (ankle, knee and hip) were calculated first for validation in the laboratory.

**Figure 4:**
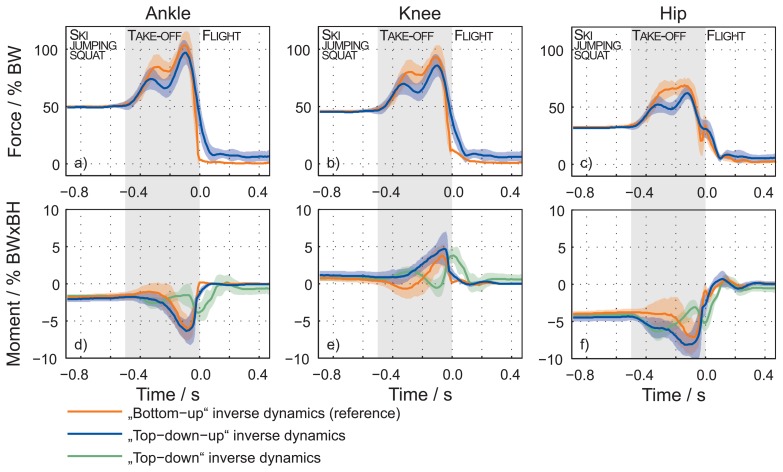
A comparison of the mean with the standard deviation of the calculated forces (**a**–**c**) and moments (**d**–**f**) in the joints according to the bottom-up, top-down and top-down-up methods during the laboratory experiment.

Graphs a–c in Figure 4 show a comparison of the calculated forces in the joints, normalized to body weight (BW), according to the bottom-up (reference) and top-down-up methods. In the initial phase, the forces match and the differences are small. During the TO phase, the root-mean-square error (RMSE) for the joint forces increase. The RMSE for forces in the ankle, knee and hip are 58.1(132) N, 54.9(123) N and 43.5(92) N. Calculated deviation (maximum error vs. reference value) for forces in the ankle, knee and hip are 9.7(14) %, 10.3(15) % and 11.2(13) %, respectively.

Graphs d–f in Figure 4 show a comparison of the calculated moments normalized to body weight and height (BW × BH) in the joints according to the bottom-up (reference), top-down and top-down-up methods. When a jumper is in the initial position, the moments according to the top-down and top-down-up methods match the reference. The differences in the moments appear during the TO phase. The top-down-up method has proven to be more accurate; the RMSE during phases are 7.5(25) N m, 9.1(23) N m and 10.9(33) N m, respectively, for moments in the ankle, knee and hip. The calculated deviation for moments in the ankle, knee and hip are 11.2(37) %, 15.6(24) % and 11.2(21) %, respectively. The RMSE and calculated deviation moments for the top-down method are much bigger; leading to the conclusion that the top-down method in our case is useless.

**Figure 5:**
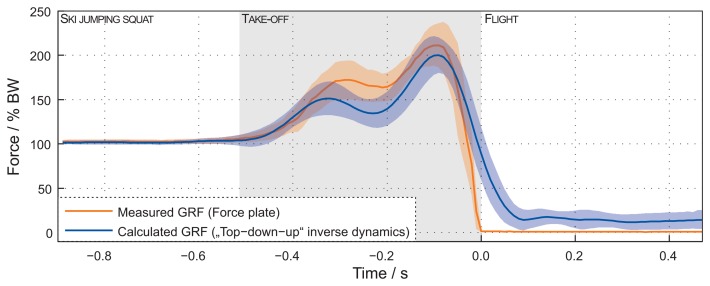
A comparison of the mean with the standard deviation of the calculated ground reaction force by the top-down-up method and the measured force with a force platform during the laboratory experiment.

As a second verification, Figure 5 shows a comparison of the calculated ground reaction force using the top-down-up method and the measured force by the force platform in the laboratory. Forces are normalized to the body weight (BW). In the initial phase, the forces match and the differences are small. During the TO phase, the differences increase. The RMSE is 65.2(259) N, and the calculated deviation is 9.7(14) %.

**Figure 6:**
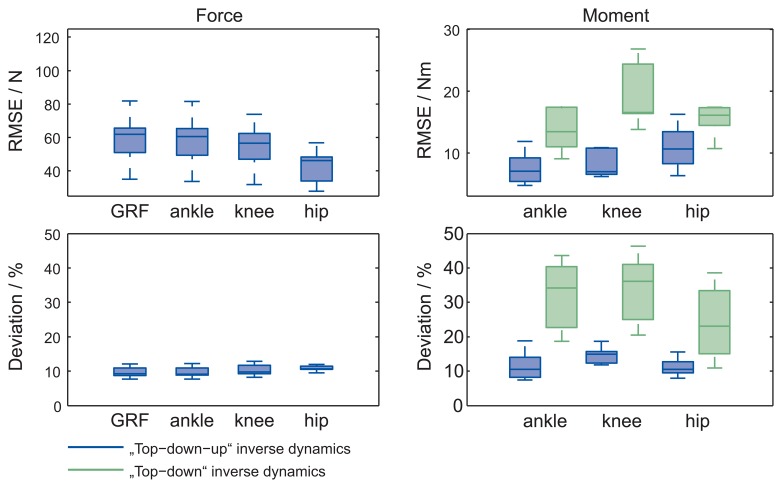
RMSE and calculated deviation for joint forces, GRF and joint moments for top-down-up and top-down inverse dynamics based on the reference value. Measurements were performed in the laboratory on a force platform. The middle line, the bottom and the top of the box present the median, 25th and 75th percentiles, respectively. The whiskers present the furthermost value in the 1.5 interquartile ranges.

Further analysis for the laboratory experiment is made for data in Figures 4 and 5 by calculating the RMSE and the calculated deviation for forces and moments (Figure 6). Here it is evident that error accumulates as the recursive procedure runs down the calculation chain. For top-down-up inverse dynamics, the maximum calculated deviation joint forces are in the 12% range, and joint moments are in the 20% range of the reference value.

### 3.2. Outdoor Validation Results

**Figure 7:**
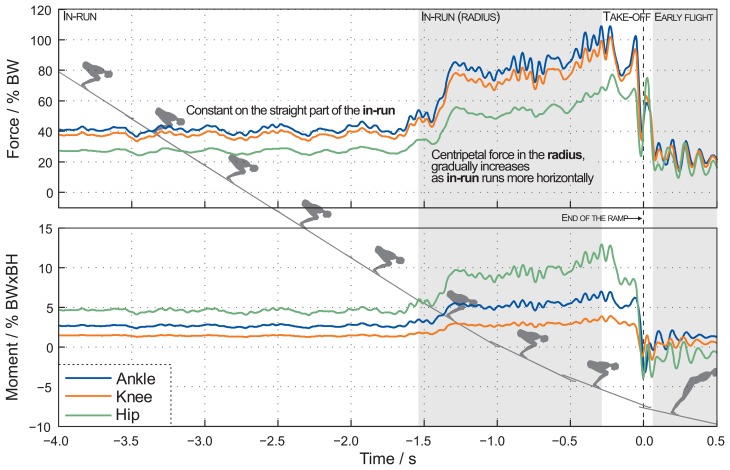
A comparison of the calculated forces and moments in the joints according to the top-down-up method during a ski jumping training session. The values represent one jump.

For the jumps on the ski jumping hill, the forces and moments in the joints (ankle, knee and hip) are calculated with the top-down-up procedure. The measurement protocol is described in a previous section. Figure 7 shows a comparison of the calculated forces and moments in the joints, 4 s before and 0.5 s after the end of the table. When the jumper is in the straight part of the IR phase, before the curved part of the ski jumping hill, the forces normalized according to the ski jumper's equipment and body weight (BW), and the moments normalized to the ski jumper's body weight and height (BW × BH) in one ankle, knee and hip are constant. Centripetal force in the radius gradually increases as the IR runs more horizontally.

**Figure 8:**
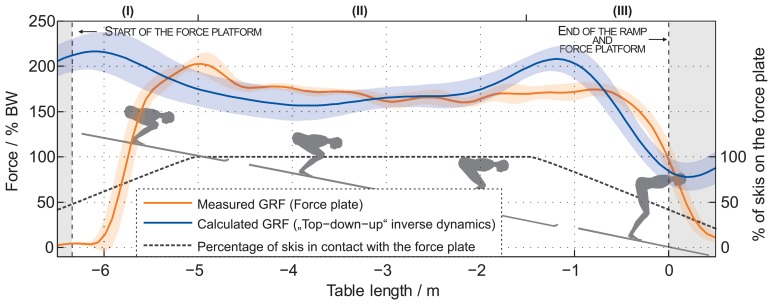
Comparison of the mean with the standard deviation of the calculated GRF and measured force with the built-in force platform according to the top-down-up method during the outdoor experiment.

**Figure 9:**
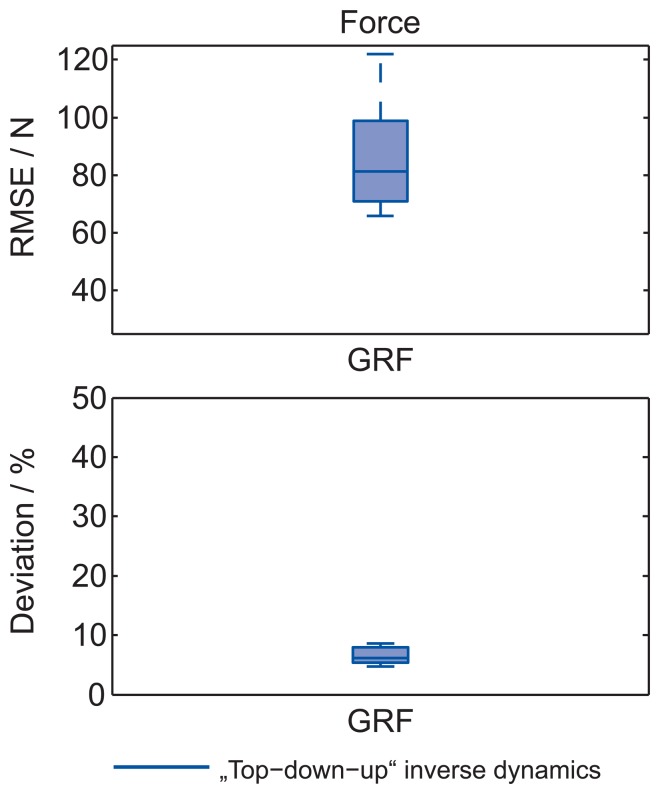
RMSE and calculated deviation of GRF for the top-down-up inverse dynamics based on the reference value. Measurements were performed during the outdoor experiment on the ski hill. The middle line, the bottom and the top of the box present the median, 25th and 75th percentiles, respectively. The whiskers present the furthermost value in the 1.5 interquartile ranges.

Figure 8 shows a comparison of the measured forces with the built-in force-platform on the table and the calculated GRF by using the top-down-up method in the last 6.5 m of the ski-hill and 0.5 m of the EF. Forces are normalized to BW. The analysis of the calculated GRF shows that a jumper produces a force of 171.6(155) % of BW during the TO phase. The analysis of the measured force with the built-in force-platform shows that a jumper produces a force of 171.3(84)% of BW during the TO phase. The difference in the shape of both forces is commented on later in the discussion. The RMSE and calculated deviation of the calculated and measured (reference) forces are presented in Figure 9. The RMSE of GRF is 81.3(184) N. The calculated deviation is 6.1(14) %.

## 4. Discussion

The main objective of this work is to provide insight into the body joint reaction forces and moments during IR and TO phases, as well as to calculate the GRF during the TO phase of a ski jumper, based on miniature wearable IMU sensors and modeling. The whole study contained three parts. Firstly, the kinematic data measured with the IMU were compared to reference data measured by the Optotrak system during the laboratory experiment. Secondly, the same data served as the input set into the dynamic model, which relies on the Newton–Euler inverse dynamics analysis. Each segment of the jumper is treated as an independent body. The recursive calculation required by Newton–Euler analysis can be made in three different sequences, as explained in the Methods, shown in the Results and discussed further in this section (bottom-up, top-down-up and top-down methods). The laboratory experiments, which provided direct GRF measurement with a force plate, demonstrated that use of top-down-up leads to the smallest errors in the forces and moments acting on the individual joints of the body segments. The third part of this work was the implementation of this method with the utilization of IMUs for jumps on the ski jumping hill, the results of which are presented in Figures 7 and 8. The current state-of-the-art ski jumping hills implement a built-in force platform on the ski jump table. GRF that measured by such a platform and GRF that is calculated from data, collected with IMUs, by the top-down-up method, are finally compared.

Kinematic measurement: The kinematic measurement method, based on a wearable IMU-based system, gave an accurate estimation of the kinematic parameters of each joint while performing the ski jump. The results of IMU kinematics in the laboratory tests were convincingly close to those of the reference Optotrak system, presenting small errors in the mean and SD of the signal. This confirmed that the wearable measurement system is suitable for use and adequate in accuracy for the ski jump analysis. This has previously been demonstrated by Chardonnens *et al.* [10], who measured the orientations of the segments with IMUs and compared traces to other studies that used other possible techniques of measurements. The difference was less than 6° for 75% of jumps and less than 15° for 95% of jumps, confirming the validity of the IMU method.

Laboratory experiment: The results of different methods applied in a laboratory study were close during the initial phases, when a ski jumper was in an aerodynamic squat position. This is demonstrated with small errors of the signal; RMSE for this phase was less then 14 N (Figure 4). The calculated forces during the whole sequence were very much the same and presented small errors in the mean and SD values of the signals. Deviations are smaller than 12% of the measured value for the forces calculated with the top-down-up method. The calculated moments, based on different methods, differ from each other during the TO phase. During the laboratory experiment, a bottom-up method was used, where the external forces were known and measured with a force platform to obtain a reference. The top-down method does not have information about the external forces, while the method bottom-up has this information; therefore, the likelihood of an accurate outcome of the bottom-up method is increased. However, the goal was to create a method that does not require knowledge of the external forces. This goal is achieved with the third method, the top-down-up. The results of the top-down-up method were demonstrated to be closer to the reference method than the top-down method; this is shown in Figure 4. All RMSE and calculated deviations for forces and moments are given in Figure 6 for the top-down-up and top-down methods. Therefore, for further analysis of the kinetic parameters the calculation based on the top-down-up method was performed. Confirmation of the top-down-up method was the main finding of the laboratory validation study.

Ski jumping hill experiment: The calculated forces and moments in the joints according to the top-down-up method, during the IR and TO phases of a ski jump, show: (1) the mean force normalized to BW in one ankle, knee and hip increases by a factor of 1.97, 1.96 and 1.92 in the radius; (2) the mean moment normalized to BW × BH in one ankle, knee and hip increases by a factor of 2.00, 2.00 and 2.02 in the radius. This is expected, considering the simulation study reported by [5]. During the curved part, from entrance to exit, slight increases in the kinetic parameters can be observed in Figure 7. The centripetal force in the radius gradually increases as IR runs more horizontally. In the model used on ski-jumping hill, we assume that no external forces act on the HAT segment. In reality, a drag force contributes on every segment of the body including the starting segment of the chain (HAT). The purpose of ski jumper aerodynamic squat position is to minimize the drag force during IR and TO phases. Consequently, we assume that this drag force is too small to be worth consideration and needs to be further explored in the future.

Another objective of this study was also to calculate the GRF at TO following measurements with the IMU-based system as input to the dynamic model. The comparison of the results obtained by the top-down-up method for GRF calculation and direct measurements on the force platform during the laboratory experiment (presented in Figure 5) confirms the validity of such GRF calculation, as well as the adequate sensitivity of the system, which enables its use on the ski jumping hill. Finally, good fitting of the measured and calculated GRF in the outdoor experiment provides evidence that the estimation of the GRF is possible with the IMU-based system on any ski jumping hill. It is important to note that the use of the force platform on the table enables the measurement of GRF only in the part of the ski jumping hill where the platform is installed. Being an important practical advantage, the presented IMU-based system provides all of the parameters along the whole ski jumping hill.

When observing the GRF traces in Figure 8, measured and calculated with our method, three sections can be noticed: (I) the section on the left side up to −5 m; (II) the middle section from −5 m to −1.5 m; and (III) the section on the right side past −1.5 m. In the middle section (II), the orange and blue bold traces, and the lighter colors (standard deviation) are well within 10% of the measurements of the force platform and IMUs. Newton law's should not be forgotten when interpreting Sections (I) and (III). The ski jumper with skis is acting as an active force, while the ski track of the table provides the reaction force, which is distributed along the entire length of the skis. As a simplification, it could be assumed that the forward and backward half of the skis could each provide 50% of the reaction force. While the jumper approaches the force platform built into the table of the ski jumping hill, the skis gradually slide on the force platform. This causes an increasing portion of the reaction force to contribute to the force platform reading, which simultaneously decreases the amount of reaction force that is still supported by the ski track before the −6 m, where the force platform starts. Consequently, the force platform reading does not provide the entire GRF value immediately, but the reading first rises, while the portion of skis in contact with the force platform increases. The action-reaction gradual loading effect in the starting part of Figure 8 might indicate that our method offers a more realistic GRF value for this region compared to the force platform. The method, however, still needs to be further explored and verified in the future.

In Section (I), the initial overshot measured with the force platform at −5 m is only a delayed effect of a longer event, which appeared before the beginning of the force platform. A very similar situation can be observed in the section on the right side past −1.5 m. In Section (III), the jumper is leaving the table and force platform, which is less and less loaded, and consequently, only a portion of the reaction force is measured. The missing overshot is not measured with the force platform at −1.5 m, because the jumper starts leaving the table.

Further support for the validity of the IMU and dynamic modeling can be found with a comparison of Figure 5 (a jump recorded in the laboratory experiment), Figure 7 (a jump recorded on the ski jumping hill) and Figure 8 (GRF calculated from jump data recorded on a ski jumping hill). A similarity between the measured GRF and the calculated GRF can be observed in Figure 5 (within the time −0.25s to 0.00 s). However, this similarity is not observed between measured and calculated GRF on the ski hill (Figure 8). A similarity between the calculated GRF from Figure 5 and calculated GRF from Section (III) in Figure 8 can be noticed. However, this similarity is not evident between measured GRF from Figures 5 and 8.

## 5. Conclusions

IMU-based measurement of ski jumpers' movements and joint load is a promising tool for trainers and researchers to better understand the kinematic parameters and joint dynamics during a ski jump. It is easy to use and does not need a team of expert staff to operate on any ski jumping hill size and place. In the indoor and outdoor experiments, the system was able to indirectly provide the values of forces and moments in the joints of the ski-jumpers' body segments, as well as the GRF during the IR and TO phases. In the future, the system could be extended to analyze the kinetic parameters during flight and landing.

## Figures and Tables

**Table 1:** Description of parameters of Newton–Euler inverse dynamics analysis.

*c_i_*	center-of-gravity of coordinate system of segment *i*
*m_i_*	total mass of segment *i*
**I***_i_*	inertia tensor matrix of segment *i* about the center-of-mass of segment *i*
***r***_*c_i_,i*_,***r***_*c_i_,i*+1_	vector from the center-of-gravity of segment *i* to joint *i* or *i* + 1
**p̈**_*S_i_*_	acceleration of the center-of-mass of segment *i* with taking into account the gravity acceleration
***ω****_i_,* ***ω̇****_i_*	angular velocity and acceleration of the *i*-th coordinate frame
**f***_i_*	joint reaction force at the segment *i*
***μ****_i_*	joint torque at the segment *i*

## References

[1] Schwameder H. (2008). Biomechanics research in ski jumping, 1991–2006. Sports Biomech..

[2] Jošt B. (2009). Teorija in Metodika Smučarskih Skokov: (Izbrana Poglavja).

[3] Virmavirta M., Kivekäs J., Komi P.V. (2001). Take-off aerodynamics in ski jumping. J. Biomech..

[4] Virmavirta M., Isolehto J., Komi P., Schwameder H., Pigozzi F., Massazza G. (2009). Take-off analysis of the Olympic ski jumping competition (HS-106m). J. Biomech..

[5] Ettema G.J., Braten S., Bobbert M.F. (2005). Dynamics of the in-run in ski jumping: A simulation study. J. Appl. Biomech..

[6] Sasaki T., Tsunoda K., Uchida E. (1994). The effect of segment power in ski jumping. J. Biomech..

[7] Schmölzer B., Müller W. (2002). The importance of being light: aerodynamic forces and weight in ski jumping. J. Biomech..

[8] Virmavirta M., Isolehto J., Komi P., Brüggemann G.P., Müller E., Schwameder H. (2005). Characteristics of the early flight phase in the Olympic ski jumping competition. J. Biomech..

[9] Chardonnens J., Favre J., le Callennec B., Cuendet F., Gremion G., Aminian K. (2012). Automatic measurement of key ski jumping phases and temporal events with a wearable system. J. Sports Sci..

[10] Chardonnens J., Favre J., Cuendet F., Gremion G., Aminian K. (2013). A system to measure the kinematics during the entire ski jump sequence using inertial sensors. J. Biomech..

[11] Bächlin M., Kusserow M., Tröster G., Gubelmann H. Ski jump analysis of an Olympic champion with wearable acceleration sensors.

[12] Ohgi Y., Hirai N., Murakami M., Seo K. (2008). Aerodynamic Study of Ski Jumping Flight Based on Inertia Sensors (171). The Engineering of Sport 7.

[13] Chardonnens J., Favre J., Cuendet F., Gremion G., Aminian K. (2013). Measurement of the dynamics in ski jumping using a wearable inertial sensor-based system. J. Sports Sci..

[14] Kusserow M., Amft O., Gubelmann H., Tröster G. (2010). Arousal pattern analysis of an Olympic champion in ski jumping. Sports Technol..

[15] Beravs T., Podobnik J., Munih M. (2012). Three-axial accelerometer calibration using Kalman filter covariance matrix for online estimation of optimal sensor orientation. IEEE Trans. Instrum. Meas..

[16] Palermo E., Rossi S., Marini F., Patanè F., Cappa P. (2014). Experimental evaluation of accuracy and repeatability of a novel body-to-sensor calibration procedure for inertial sensor-based gait analysis. Measurement.

[17] Abramowitz M., Stegun I.A. (2012). Handbook of Mathematical Functions: With Formulas, Graphs, and Mathematical Tables.

[18] De Leva P. (1996). Adjustments to Zatsiorsky-Seluyanov's segment inertia parameters. J. Biomech..

[19] Paul B. (1979). Kinematics and Dynamics of Planar Machinery.

[20] Paul J.P. (1966). Paper 8: Forces Transmitted by Joints in the Human Body. Proc. Inst. Mech. Eng..

